# The Hampshire Tuberculosis Officership

**Published:** 1914-06-13

**Authors:** 


### THE HAMPSHIRE TUBERCULOSIS OFFICERSHIP.

There have been handed to us by a correspondent forms of application and other informatory literature relating to the above appointment, the announcement of which had already attracted our notice, because the initial salary offered (£400 per annum) is substantially below that recommended in the Report of the Royal Commission on Tuberculosis, and in force in nearly every county area which has provided itself with such an official. In view of this fact, it might have been supposed that some countervailing advantage existed; either in the duties being easier, or the conditions in one way or another more attractive. Examination of the forms above mentioned does not, however, at all bear out the supposition just mentioned. The duties and conditions of appointment are, on the contrary, more stringent than usual. It may be of profit and interest to those concerned with medical work in salaried posts under the Insurance Act to enumerate them.

In the first place, besides tuberculosis duty, general public health work may be demanded of the successful candidate; while in the event of the establishment of a county sanatorium he may be required to act as its medical superintendent. It appears that the prospect of a salary of £500, to be reached in four years, is final; £500 is the maximum. The officer is expected, too, to keep a bicycle which must be used whenever practicable; £10 a year is allowed for this, and the travelling expenses otherwise, indeed, are not unfair. The amount of travelling to be expected is deducible from a typewritten letter from the medical officer of health accompanying the other forms. The total county area is 942,000 acres: in order to reach outlying parts it may be necessary to catch early trains and to cycle several miles. Occasionally it may be necessary to be away from home for a few nights (subsistence allowance is then made). There is a certain amount of clerical work, and altogether "the work is by no means light." The duties, however, are such as will undoubtedly afford most valuable experience in tuberculosis work and all branches of public health work.

Now all this reads unsatisfactorily, in the general interest, in the interest of prospective candidates, and indeed to the credit of the Hampshire County Council both as fair-minded persons and as business men. If a tuberculosis officer for a largish county with a scattered population be wanted, why offer an inferior salary? The result in the long run is likely to be inferior work. Next, why require of him in addition work outside his own province, more especially as this body simultaneously advertises for two assistant medical officers of health? That tuberculosis work does nowadays require special experience and training is recognised in these application forms, which require of candidates particulars of such. It was also recognised a year or two ago by their present medical officer of health, who, as was then brought to our notice, circularised sanatorium superintendents with a request for information about the use of tuberculin. But if it be held out as an inducement that the work will afford most valuable tuberculosis experience, then the intention would seem to be adumbrated of appointing someone who lacks it—a class of person, indeed, who may be expected to be well represented amongst such applicants as may come forward undeterred by the liability to move suddenly to the quite uncertain residential accommodation of a municipal sanatorium.

This proposal illustrates, indeed, one of the risks incurred in giving the sanitary service charge of anti-tuberculosis work. Junior and inferior men can, of course, be attracted, but it is the sick public who suffer. For on the actual clinical diagnosis and decisions, and on the therapeutics, of the tuberculosis officer there is no check at all. He may miss all those fine early diagnostic signs which need learning and carefully practising, and no one is the wiser. He may, and indeed often does, guess at the existence or not of laryngeal disease, not being able to inspect it, and a moderate amount of prevarication pulls him (but not the patient) through. We do not think that responsible laymen, if they knew what their scheme really meant, would fail to alter it; and meanwhile we could not advise any self-respecting tuberculosis worker to enter himself as a candidate for the post of tuberculosis officer to the Hampshire County Council.

